# Obstetrics and Gynecology

**Published:** 1893-02

**Authors:** Wm. Kieller

**Affiliations:** F. R. C. S. Ed., Professor of Anatomy University of Texas; late Physician for Diseases of Women, Edinburgh Provident Dispensary; Galveston


### OBSTETRICS AND GYNECOLOGY.

[By Wm. Kieller, F. R. C. S. Ed., Professor of Anatomy University of Texas; late Physician for Diseases of Women, Edinburgh Provident Dispensary.]

TUBAL PREGNANCY.--Jos. Price, M. D., (*American Journal of Obstetrics and Diseases of Women*) states that all cases of ectopic pregnancy met by him have been primarily tubal. Ovarian pregnancy is possible, but not probable,—he considers that they rarely pass into broad ligament pregnancies.

The causes are, 1st, anatomical, e. g., absence of ciliated epithelium from previous salpingitis; 2d, moral, viz: the fear of conception in illigitimate intercourse (absent or perverted ciliary motion).

Salpingitis from any cause predisposes. Statistics of pregnancy are unreliable. His own experience in section for ectopic pregnancy is a little over 1 per 1000 pregnancies.

Cases left to themselves have the very gravest prognosis, few recovering, and many who do so recovering by suppuratic processes of the most horrible character.

The causes of death are hemorrhage, septicæmia, peritonitis, perforation of important viscera by bone. Rupture and hemorrhage cause most deaths.

The time of rupture is oftenest in first month, less so in third month, occasionally as late as fifth month.

Treatment, other than by operation, is unworthy of consideration; and operation is imperative as soon as the case has been diagnosed.

The earlier the operotion is done, the easier it is,—leaving the gestation to go on in hope of getting a living child, is fraught with too much risk to be warrantable; and the operation itself, simple in the early months, is grave in the latter months, mainly from the difficulty in removing the placenta.

The vaginal method is universally condemned, except in rare cases. Dr. Price has operated on eighty-three cases, with three deaths, as compared with statistics showing 67% of deaths in 500 cases treated expectantly.

The main risk of the operation is hemorrhage. If patient is so weak that she is not likely to bear the operation, Dr. Price transfuses saline fluid first, to restore arterial tension. The placenta should be removed in every case, or washed and hermetically sealed. Removal often taxes all the skill of the surgeon, the bleeding being profuse. Rapid separation, hot water and firm pressure, will usua'ly control the hemorrhage. A drainage tube should be used where oozing is expected.

Very few cases are cocognized till rupture takes place.

---

CASES OF EXTRA-UTERINE PREGNANCY (Ruf. B. Hall, *Am. Jour. of Obstet. and Dis. of Women*)—The following abstract of these cases illustates well the usual course of extra-uterine gestation:

1. Mrs. M., age 30; married 9 years; three children, youngest 5 years.

*Previous History.*—Abortion three years ago; septic trouble; chronic salpingitis.

*Menstrual History.*—Last normal period January 3, 1891. Returned February 1, and flowed for seven days; flow intermittent, with much pain. From February 7–12 free from pain. February 12, abdominal cramps, followed by general tenderness. February 24, fainted; great and persisting exhaustion. March 8, slowly increasing tumor in left side; uterus in front, and displaced to right, slightly enlarged; pelvic floor displaced downward; tumor firm and fixed.

*Diagnosis.*—Ectopic pregnancy, with rupture into left broad ligament.

*Operation.*—Found intra-peritoneal rupture of sac; removed it; uninterrupted recovery.

CASE 2.—Mrs. W., age 33; 2 children, youngest 15 months. Regular till the 5th of May, when she menstruated as usual. Next June 16, irregular painful flow for ten days. June 29, severe abominal pain with some collapse (probably date of rupture), followed by abdominal tenderness. July 7, examination under chloroform revealed nothing abnormal in pelvis. July 8–19, at work, then got worse. July 24, tumor size of large orange to right of uterus; temperature, 100–102°. July 27, abdominal section; suppurating extra-uterine pregnancy in retro-uterine space, shut off by adherent intestines; fæculent odor. Fæces discharged by drainage tube twenty-four hours afterwards; sinus closed October 5. Good recovery.

CASE 3.—Mrs. H., age 30; married 8 years; last child 6 years old. Last normal menstruation October 16–20. November 25, slight flow. December 16, slight flow for a few hours, then *severe pain and collapse;* improved and going about. January 5, return of pain, and marked collapse.

*Diagnosis.*—Extra-uterine pregnancy. Waited for patient to rally, and operated January 7. Found primary extra-peritoneal rupture, with one pint old and one and one-half pints recent blood in peritoneum. Surgeon believes date of rupture was December 16. Considers that he did wrong in waiting for patient to rally from January 5th to 7th. She lost more blood, and ran a greater risk.

CASE 4.—Age 33, married 9 years; one child 8 years ago, second 16 months ago. Last normal menstruation middle of September, 1891. Reappeared November 15, and lasted one week; disappeared a week, and reappeared, and continued till operation. Abdominal cramps, pain, and collapse on December 21, followed by abdominal tenderness. On January 9, pulse 130; temperature 103°. History of sepsis for fifteen days; yellow skin.

*Physical Examination.*—Tumor in left abdomen and pelvis, size of adult head; pelvic floor depressed; tumor semi-solid.

*Diagnosis.*—Extra-uterine gestation.

*Operation.*—Organized blood clot; fæculent odor; four or five pints recent blood clot; gestation sac formed by left tube; no fœtus found; all removed, irrigated and drained. Patient recovered, with fæcal fistula, which remained open for three weeks, and then closed.

NOTE.—Physical examination suggested subperitoneal rupture; operation showed that it was from the first intra-peritoneal.

CASE 5.—Mrs. C., age 32; last child 7 years old. History of pelvic trouble for three or four years; worse last seven months. Pain in ovarian regions. Intensified pain in left ovarian region from the end of May. June 10, mass the size of a large orange to the left of uterus; right ovary adherent.

*Operation* commenced for undefined condition: unruptured ectopic pregnancy at about five weeks, found and removed. Right ovary and tube adherent—removed. Good recovery.

CASE 6.—Mrs. S., age 24; married five years; no children. Pelvic trouble since marriage; much worse during past two weeks. Menstruation a few days late, and lasted ten days; freer than usual.

*Physical Examination.*—Left ovary adherent. Right ovarian region extremely tender; indistinct bagginess, but no defined mass could be made out. Three weeks later nothing distinct could be felt, but as she was evidently losing strenght, operation was advised, no diagnosis having been made.

*Operation.*—Removal of three pints blood clot from peritoneal cavity, and a ruptured gestation sac containing decidua and formed by left tube; opposite ovary and tube being adherent, also removed. Irrigation and drainage; recovery.

Dr. Hall draws the following conclusions:

1. It is impossible to diagnose with certainty whether rupture has taken place into the peritoneal cavity or between the layers of the broad ligament, especially in the earlier months. Therefore, treat all these cases as if certain that the rupture is intra-peritoneal.

2. In early intra-peritoneal rupture, bleeding may go on slowly into the peritoneal cavity for weeks, the more fluid part of the blood being absorbed, and the size of the tumor giving no indication of the amount of blood lost.

3. Adhesions of intestines of only four or five weeks duration may compress the clot, and markedly depress the pelvic floor.

4. It is not always easy to diagnose ectopic pregnancy in the early weeks.

5. Abdominal section is imperative, as soon as a diagnosis has been made.

ON SOME CASES OF ECTOPIC GESTATION (J. W. Taylor, F. R. C. S., *British Gynecological Journal*, August, 1892)—Mr. Taylor considers that ectopic pregnancy is almost always primarily tudal, but finds that its further course is extremely variable, and quotes cases in illustration:

1. A case in which there was free hemorrhage from the fimbriated end of the tube, and irregular uterine hemorrhage without rupture of the tube. On operation, a mole was found in the ampulla.

2. Case 2 had the usual symptoms to tubal gestation, which had ruptured five weeks after last normal mestruation. Collapse was very marked. On operation, much fluid blood escaped from the peritoneum. The tube held a small ovum, which protruded from a slight rupture. It was interesting from the smallness of the lesion which caused such profuse hemorrhage, and further, because the tube showed a cicatrix of a previously ruptured gestation sac, associated with a history two years previously, of pelvic hæmatocele, from which she recovered without operation.

3. Case 3 gave a history of regular menstruation, followed by two months amenorrhæa; then pain and menorrhagia, with an increasing tubal tumor. An unruptured tubal gestation was removed at the fourth month. Placental structure, but no trace of fœtus, were found.

4. Case 4. Previous regular menstruation; no distinct history of amenorrhæa; then irregular uterine hemorrhage, with pain and increasing tumor on the right side. On operation, a four months ovum, with fully developed fœtus, was found; the placenta was attached to the fimbriated end of the tube, and the gestation sac was formed by dilated tubal extremity and adherent omentum, the latter roofing in the sac and separating it from the general peritoneal cavity.

5. Case 5 was one in which a living, full-time fœtus was found free in the peritoneal cavity, the membrane being represented by a fringe around the placenta. The placenta was attached to the right iliac fossa and right side of the true pelvis. It was removed with the child. Mother recovered, and child lived some months after birth.

6. Case 6 gave a history of previous regular menstruation, followed by one year's amenorrhæa; increasing abdominal tumor; uterus enlarged to five inches, and displaced forward and to right; abdominal tenderness, toward the end, very great; and in the latter months, pyrexia, rigors, vomiting, and prostration. Diagnosis was extra-uterine gestation, beyond full term, with dead fœtus, or ovarian tumor. Operation revealed absence of general peritonitis. Behind the omentum, but attached to it by slight adhesions, was a thick walled sac containing dirty brown fluid, a decomposed full time fœtus, and placenta. Placenta left to slough away, and sac stitched to abdominal wall, and drained. Recovery.

7. Case 7 had a history of six weeks amenorrhæa, then symptoms of peritonitis with a tumor, evidently a hæmatocele on left of uterus. Diagnosis, probable ruptured extra-uterine gestation; hemorrhage limited by adhesions. Six weeks rest produced absorption of the hæmatocele. *The diagnosis was proved correct* by the appearances discovered two years subsequent, when operation was undertaken for a second extra-uterine gestation in the same tube, which this time almost cost the patient her life, by the profuse hemorrhage.

Mr. Taylor considers these cases interesting, as affording evidence:

1. Of hemorrhage without rupture of the tube. (Bland and Suttons Incomplete Tubal Abortion.)

2. Of recurrent ectopic pregnancy, with rupture in both cases.

3. Of marked and persistent enlargement, without rupture.

4. Of diagnosis before rupture.

5. Of cure without operative treatment.

6. Of the possible stages through which a tubal pregnancy may become abdominal.

Taking these cases in conjunction with a specimen in Queen's College Museum, Birmingham, he concludes that:

1. All tubal pregnancies are originally tubal.

2. They may rupture between the layers of the broad ligament, and develop there (extra-peritoneally).

3. The sac, partly composed of fallopian tube, partly of broad ligament, may expand upward into the abdominal cavity, forming an abdominal cyst.

4. The child may develop free in the abdominal cavity, covered by errosion or naked; the placenta being attached in the pelvis, and partly to the tube.

These abstracts of cases I have given pretty fully, because they afford typical clinical histories of extra-uterine gestation. Taken with the folllowing, and with the preceding abstract of

Dr. Price's paper, they furnish a good resume of the most advanced views as to the diagnosis and treatment of a condition which every general practitioner should be thoroughly familiar with.

In a paper read before the Edinburgh Obstetrical Society last year, Dr. Halliday Croom concluded that on the following four grounds, a case of extra-uterine gestation, if seen early and watched carefully, can be diagnosed *before rupture of the sac* with a very great amount of precision:

1. The general signs of pregnancy,—for example, the cessation of the menses.
2. The displacement of the uterus to one side by a tumor, which gradually grows.
3. The passage of the decidua, in whole or in part, and irregular hemorrhages.
4. The presence of paroxysmal pain localized to one side, though not to one spct.

All those signs must be present, but, in diagnosis before rupture, the essential point is the passage of the decidua.

NOTE.—In all cases where ectopic pregnancy is suspected, all blood clots should be examined by floating out in water, for decidual shreds.

